# Novel Handheld Magnetometer Probe Based on Magnetic Tunnelling Junction Sensors for Intraoperative Sentinel Lymph Node Identification

**DOI:** 10.1038/srep10842

**Published:** 2015-06-03

**Authors:** A. Cousins, G. L. Balalis, S. K. Thompson, D. Forero Morales, A. Mohtar, A. B. Wedding, B. Thierry

**Affiliations:** 1 Ian Wark Research Institute, University of South Australia, Mawson Lakes, SA 5095, Australia; 2 Discipline of Surgery, University of Adelaide, Adelaide, SA 5000, Australia; 3 School of Engineering, University of South Australia, Mawson Lakes, SA 5095, Australia; 4 Medical Device Research Institute, School of Computer Science, Engineering and Mathematics, Flinders University, Bedford Park, SA 5042, Australia

## Abstract

Using magnetic tunnelling junction sensors, a novel magnetometer probe for the identification of the sentinel lymph node using magnetic tracers was developed. Probe performance was characterised *in vitro* and validated in a preclinical swine model. Compared to conventional gamma probes, the magnetometer probe showed excellent spatial resolution of 4.0 mm, and the potential to detect as few as 5 μg of magnetic tracer. Due to the high sensitivity of the magnetometer, all first-tier nodes were identified in the preclinical experiments, and there were no instances of false positive or false negative detection. Furthermore, these preliminary data encourage the application of the magnetometer probe for use in more complex lymphatic environments, such as in gastrointestinal cancers, where the sentinel node is often in close proximity to other non-sentinel nodes, and high spatial resolution detection is required.

In the Tumour Node Metastasis (TNM) classification system, the lymphatic system plays a central role in the standard of care for breast cancer and melanoma. Of most prognostic significance is the sentinel lymph node (SLN): the first-tier lymph node to receive drainage from a primary tumour. The presence of metastasis in the SLN has a strong prognostic value in a number of solid cancers^1^,^2^,^3^. If the SLN can be accurately identified and distinguished from surrounding or lower-tier nodes, then a much more targeted approach to cancer staging and treatment is achievable. In order to identify the SLN, lymphotropic contrast agents must be used, as the ‘sentinel’ status cannot be determined based on anatomical features or location alone. Currently the gold standard lymphotropic contrast agents for breast cancer and melanoma are low soluble blue dyes and radioactive colloids (usually ^99m^Tc-based). The role of the dyes is to visually guide the surgery by staining the lymphatic vessels leading from the tumour site and subsequently, stain the SLN. Radioactive tracers have the advantage that gamma camera or SPECT imaging can be performed preoperatively to provide the surgical team with a lymphatic ‘roadmap’. During the surgery, the location of the SLN can then be confirmed with a handheld gamma-detecting probe. Both contrast agents may be used independently, but greater accuracy can be achieved if they are used simultaneously^4^. Whilst these gold standard contrast agents have well-established efficacy in breast cancer and melanoma SLN identification, they have limited application in cancer types with more complex lymphatic draining routes, due to radiation shine-through affecting spatial resolution and accuracy.

In order to overcome the challenges associated with applying the SLN concept beyond breast cancer and melanoma, alternative approaches are currently being investigated^4^. Due to the high quality magnetic signal and validated use in clinical trials^5^,^6^,^7^, iron oxide nanoparticle-based magnetic tracers show great promise as a real-world alternative to conventional lymphotropic contrast agents. One particular advantage to using magnetic tracers is that the SLN detection protocol remains analogous to the protocol currently performed using radioactive tracers (preoperative roadmap imaging and detection with a handheld probe). Furthermore, magnetic tracers eliminate the need for ionising radiation sources and therefore reduce the logistical issues currently associated with the manufacturing, handling, and licensing required to administer radioactive tracers. In the magnetic tracer alternative, magnetic resonance imaging (MRI) can be readily used instead of gamma cameras for preoperative localisation of the SLN, providing high-resolution imaging and consequently better anatomically detailed images compared to gamma cameras.

To enable intraoperative detection of the SLN, handheld magnetometer probes are required in place of gamma probes. A number of magnetometer probe technologies have been proposed using a range of sensing technologies – from Hall effect sensors^8^,^9^,^10^ and SQUID sensors^11^, to the current embodiment of a commercially available instrument, the SentiMag probe, which uses precisely constructed induction coil sensors^12^. The SentiMag probe provides a notable example of the application of magnetic tracers and magnetometer detection of sentinel nodes and has been demonstrated in a large clinical breast cancer trial^6^ and recently, in a clinical prostate cancer trial^7^. A common compromise that exists with conventional magnetometer technology (Hall effect, giant magnetoresistor, fluxgate, induction coil, or SQUID based sensors) is that cost and sensitivity are often inversely related. For example, whilst some commercial Hall effect sensors can be bought for less than $1, their sensitivity is often limited to the millitesla range. Alternatively, the cost for SQUID magnetometers can extend into hundreds of thousands of dollars, which is a reflection of their superior (femtotesla) sensitivity.

Magnetic tunnelling junction (MTJ) sensing technology is as an ideal compromise between cost and sensitivity. Limited by the nanofabrication techniques required to produce them, MTJ sensors were not developed until the early 1990s, with commercial products available in limited supply from the early 2000s. MTJ sensors utilise the spin tunnel magnetoresistance phenomenon, with passive electrical characteristics dependent on the net magnetic flux parallel to the sensor’s sensing axis. Like Hall effect and giant magnetoresistor sensors, they can be integrated into surface-mounted packages for electronics, or as bare die can be used to make magnetometers with a sensing area only tens of microns wide^13^ In the last 15 years, development of MTJ technology has been rapid, with room temperature tunnelling magnetoresistance (an indicator of MTJ sensitivity) increasing from approximately 40**%**^14^ to over 600%^15^ Currently available commercial MTJ sensors, such as the STJ-240 (MicroMagnetics, Massachusetts, USA) can be bought off-the-shelf with room temperature magnetoresistance as high as 200% and capable of measuring nanotesla (nT) fields, yet (at the time of writing) only cost US$50. With such developments, magnetometers developed using MTJ technology have the potential to create an economically competitive alternative to conventional induction coil and, in particular, fluxgate magnetometers.

Importantly, MTJ technology also has the potential to offer higher spatial resolution in the detection of small accumulations of magnetic tracers. In order to distinguish individual lymph nodes in tightly packed clusters, the overall spatial resolution of handheld probes must be less than the average distance between nodes (often taken as <25 mm for gamma probes used in breast cancer and melanoma procedures^16^). Overall spatial resolution (i.e. for gamma probes) is measured as the full width half maximum (FWHM) of a device’s response curve when scanned laterally over a point source. Due to the radiant nature of gamma tracer signals and the size of detectors used in gamma probes, spatial resolution of commercial probes may vary from 13 – 20 mm depending on the manufacturer^17^ While satisfactory for detecting the SLN in breast cancer and melanoma, spatial resolutions of this order become problematic in complex lymphatic environments such as oesophageal cancer, where approximately 90% of the SLNs are within 30 mm of the primary tumour^18^ and are often clustered in close proximity to other non-sentinel nodes.

Taking these limitations into account, a novel magnetometer probe designed using advanced MTJ sensors has been developed to enable the accurate and reliable detection of small quantities of magnetic tracers in the SLN. More specifically, the characteristic features of MTJ sensors are used here to create a sensitive, high spatial resolution, and cost-effective alternative to other magnetometer designs aimed at replacing gamma probe technology.

## Results

### Constructing the magnetometer probe

**Figure 1:**
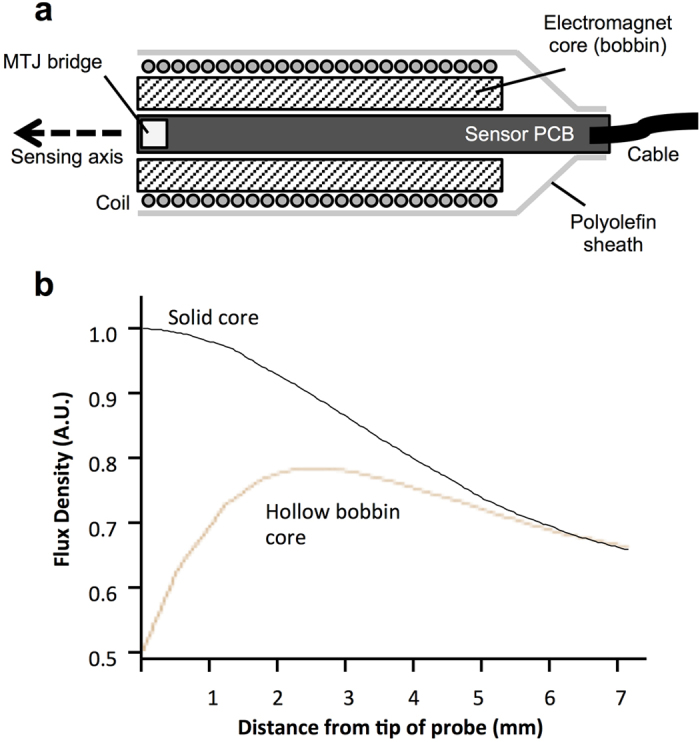
MTJ magnetometer probe schematic diagram. (**a**) The MTJ sensor bridge is mounted on the end of a small double-sided PCB, and positioned inside a cylindrical electromagnet’s aperture, forming the probe tip. The electromagnet consisted of a 90-turn coil wound onto a ferrous core and was used to excite magnetic nanoparticles in either parallel or antiparallel magnetisation with respect to the sensing axis of the probe. (**b**) Due to the hollow core of the bobbin-shaped electromagnet, from finite element analysis, the magnetic signal can be seen to peak at approximately 2.5 mm from the probe tip, before decaying in a manner similar to that of solid-core electromagnets.

A custom double-sided printed circuit board (PCB) was designed to make the probe’s sensor, and to fit securely inside the hollow core of a bobbin-shaped electromagnet. For the construction of the magnetometer probe sensor, 4 single-axis MTJ sensors (two sensors per side of the circuit board) were arranged into a full-bridge Wheatstone configuration on one end of the PCB. The sensor bridge had a total resistance of 1.5 kΩ (in zero field) with an input current of 3.3 mA. The sensor PCB was mounted inside a 90-turn cylindrical electromagnet with ferrous core, which formed the tip of the probe (Fig. 1a). From finite element analysis of the electromagnet’s hollow-core bobbin design, the magnetic flux density peaks in magnitude approximately 2.5 mm from the tip of the probe, before decaying in a manner more comparable to solid-core electromagnets (Fig. 1b).

The electromagnet at the tip of the magnetometer probe was used to excite the magnetic tracer with an alternating parallel/antiparallel magnetic field (square wave alternating current, 180 Hz, 50% duty cycle). This approach was chosen such that the magnetisation signal from the magnetic tracer (in phase with the electromagnet) could be distinguished from background signals such as the baseline magnetic signal from the electromagnet, 50 Hz mains power noise, and Earth’s background magnetic field, which are often orders of magnitude greater than the magnetic tracer signal. By processing the Wheatstone bridge signal using high gain, high Q active filters, any signals not in phase with the switching electromagnet (i.e. the noise) could be strongly attenuated. Finally, the filtered signal was converted to a DC signal proportionate to the magnitude of the signal from the magnetic tracers; hence changes in the magnetometer probe output could indicate both the presence and quantity of magnetic tracer in the vicinity of the probe tip.

### Measuring the magnetometer probe detection limit

In order to measure the detection limit, a series of magnetic phantoms were made by drying various quantities of a magnetic tracer stock.

**Figure 2:**
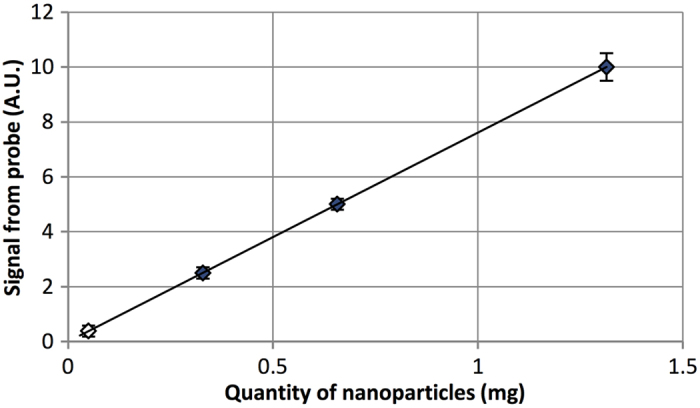
Probe limit of detection. By measuring a range of magnetic tracer quantities at a fixed distance of 4.0 mm from the signal source, **a** 50 μg limit of detection (SNR = 2.0) was calculated. Due to the short-range nature of magnetic fields, this value can be significantly improved if the probe is moved closer to the signal source. 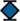 = Measured data; 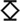 = Calculated datum.

The detection threshold was defined as the quantity of magnetic tracer detected by the probe with a signal to noise ratio (SNR) of 2.0. Detection threshold measurements for the sensor bridge were performed using three phantoms of varying magnetic tracer quantity (0.3 mg, 0.7 mg, and 1.3 mg). A custom rotating stage was used to measure multiple phantoms in sequence and control the distance between the probe and phantoms between measurements. The probe was mounted vertically above the sample stage, and positioned such that the sensor bridge was 4.0 mm from the dried particles of each passing phantom. This distance value was chosen as a practical probe-to-node distance due to the likely presence of adipose tissue or adventitia surrounding SLNs. Based on the magnitude and noise level of signal for the three measured phantoms, a 50 μg detection threshold at 4.0 mm distance was calculated (Fig. 2).

### Longitudinal range measurement

**Figure 3:**
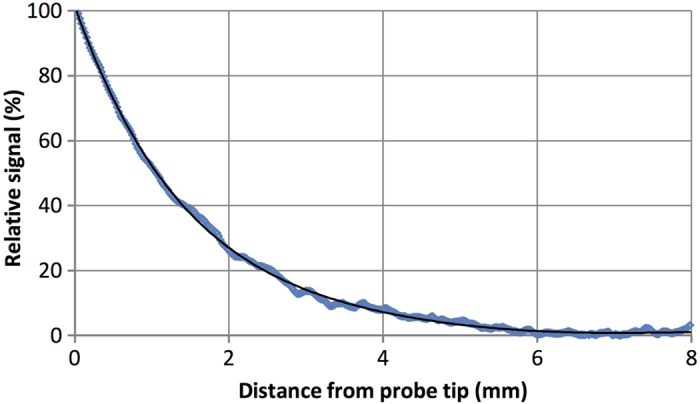
Range of magnetic signal. The magnetic signal from a simulated lymph node approximately 6 mm in diameter decays at a lower rate than predicted by theoretical inverse-cubed law for magnetic dipoles due predominantly to the finite volume of the phantom.

Longitudinal sensitivity of a device is the ability to resolve the presence of a signal at distance, and hence is a measurement of the signal from a source as it is moved further away from the tip of the probe. The MTJ magnetometer probe longitudinal sensitivity was measured using a concentrated 10 mg phantom (to improve signal to noise ratio), which consisted of 0.1 mL of colloidal tracer in a 1.5 mL microcentrifuge tube. A liquid phantom was selected as it better represented the volume of a small lymph node, compared to the thin film of tracer in dried phantoms used in detection limit measurements. The probe was mounted horizontally, and displacement (0.0 mm to 7.5 mm from the probe tip) was controlled via a 2-inch translational stage driven by a DC motor. The phantom was then mounted horizontally on one end of a 30 cm rigid plastic strip with the other end attached to the stage, to act as a spacer and attenuate any magnetic interference from the stage / motor that may be measured by the probe. From the measured data (Fig. 3), it can be seen that the magnetic signal from the phantom has a short range, with the signal decaying to 50% at a distance of just 1.0 mm, and below a SNR of 2.0 at approximately 5.1 mm.

### Lateral response curve measurement

**Figure 4:**
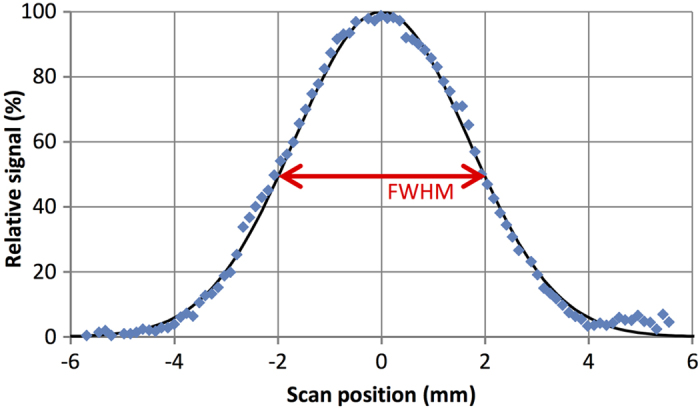
Spatial resolution of probe. Normalised response curve of a point-source phantom (centred at x = 0) scanned at a height of 1.0 mm. A probe spatial resolution of 4.0 mm can be determined from the response curve’s FWHM, which is considerably less than that of a coil magnetometer or gamma probe.

For conventional gamma probes, the spatial resolution is a measure of the lateral response of the probe, i.e. the FWHM of the signal obtained by scanning a small point source phantom past the probe at a fixed distance. Likewise, the spatial resolution of the magnetometer probe was quantified by measuring the response curve resulting from scanning a small 1.0 mg dried-tracer phantom. The translational stage was again used to provide controlled displacement of the phantom. The probe was mounted vertically during measurements such that phantom displacement was perpendicular to the sensing axis of the sensor bridge. Due to the high attenuation of magnetic signals with distance, the phantom-probe distance was reduced to 1.0 mm (longitudinal distance), and scanned ± 6.0 mm (lateral distance) from the centre of the probe (x = 0). From the resultant lateral response curve (Fig. 4), a spatial resolution of 4.0 mm for the magnetometer probe was measured.

### *Ex vivo* measurement of swine lymph nodes

**Figure 5:**
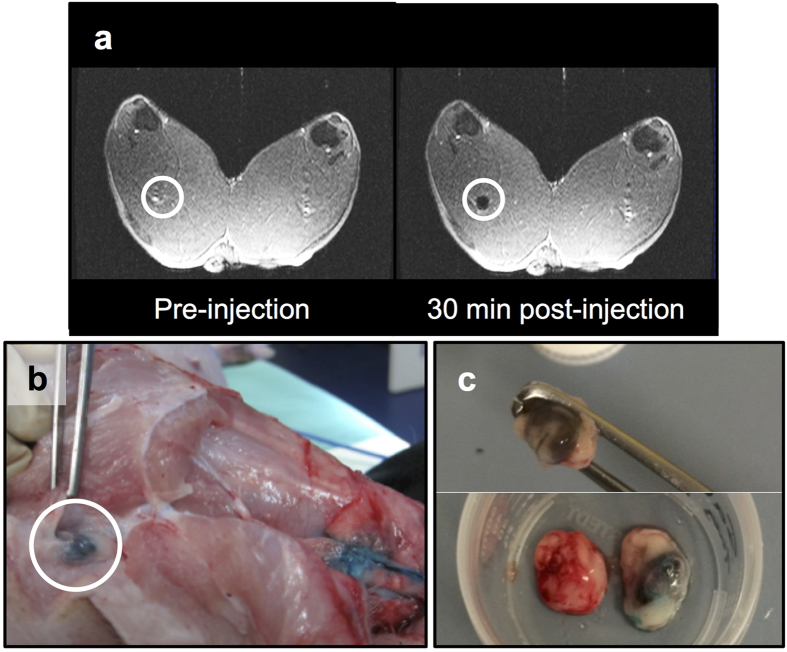
‘Sentinel’ node identification *in vivo*. (**a**) Preinjection and postinjection coronal MRI scans of a swine’s hind legs showing the negative contrast in one first-tier node (circled) resulting from the uptake of the magnetic tracer. Postinjection scans were used preoperatively to identify the location and number of first-tier nodes in both limbs for each animal. (**b**) After the anatomical location of the nodes had been determined with MRI, Patent Blue V dye was used to guide the surgery, and the identification of first-tier nodes (circled). (**c**) Some nodes were only visualised due to the dark brown staining from magnetic tracer accumulation (top). Compared to a control lymph node (bottom left), either blue dye (bottom right) or magnetic tracer uptake was sufficient to distinguish the nodes from surrounding tissue.

**Figure 6:**
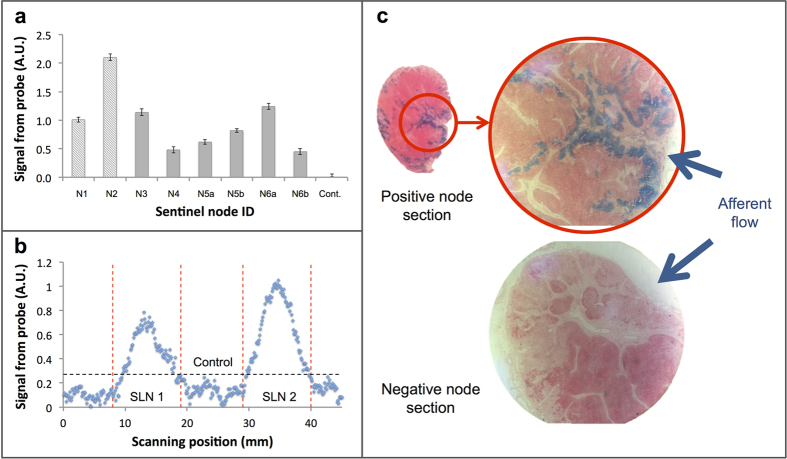
Swine lymph node measurements. (**a**) MTJ magnetometer probe measurement of first-tier nodes from three animals. N1, N2, and control (Cont.) were measured *ex vivo*, and N3 – N6b were measured *in vivo*. Error bars indicate the uncertainty in measurement due to background signal fluctuations (electronic noise). (**b**) Distinguishing nodes in close proximity in *ex vivo* array of lymph nodes. The boundary of each node is indicated with the vertical dotted lines and the threshold level is indicated with the horizontal dotted line. (**c**) Prussian blue staining of two separate node sections. The presence of magnetic tracer in afferent trabecula and subcapsular sinuses is evident in positive first- and second-tier nodes (e.g. top), but absent in negative nodes (e.g. bottom).

Evaluation of the magnetometer probe *in vivo* was determined using large animal (swine) preclinical studies. To begin with, a 10 mg quantity (20 mg/mL) of magnetic tracer was injected intradermally at a pre-marked site on the dorsal surface above each hind hoof of a single swine. T1 and T2* MRI sequences of the hind leg and groin region were acquired preinjection as a control. Postinjection, the MRI sequences were repeated at *t *= 0, 30, and 60 minutes to monitor the uptake of magnetic tracer into the first- and second-tier draining lymph nodes. Once the nodes had been identified on the MRI scans, the swine was moved to an operating theatre. Before surgery, 0.5 mL of blue dye was intradermally injected in the same marked location as the magnetic tracer. During surgery a total of 2 first-tier draining nodes (one from each hind leg) were surgically removed. Both were identified in 30 and 60 minute postinjection MRI as being deep popliteal nodes (Fig. 5a). The blue dye was used to visualise the lymphatic vessels (Fig. 5b). In one limb, the first-tier node was visibly identifiable due to the blue staining from the dye, as well as brown staining from the magnetic tracer. Conversely, the first-tier node from the other limb was only identifiable from brown staining, with no apparent uptake of the blue dye (Fig. 5c). The maximum magnetic signal from each node was found by measuring the node at various positions. Due to variations in the level of noise between measurements, SNR was favoured over outright DC signal as a cross-measurement indicator of signal strength and quality. Although both *ex vivo* nodes (N1 and N2) were of comparable size, the SNR from N1 (7.8) was smaller than that of N2 (SNR of 12.4, see Fig. 6a).

In this study, resected nodes were also used to measure the spatial resolution of the MTJ probe in a simulated close-packed node scenario. To do this, a one-dimensional array of lymph nodes (each of approximately 10 – 15 mm in largest dimension with adipose tissue and adventitia intact) was made using N1 and N2, and one negatively measured ‘control’ node (from the groin of the animal). As before, the probe was mounted vertically and the translational stage was used to control node displacement. As the uptake of tracer was non-uniform throughout the node samples (most concentrated where the primary afferent vessel entered the node), N1 and N2 were orientated such that the region of highest tracer accumulation was facing the probe. All nodes were positioned such that their top surface was 1.0 mm from the probe tip. Scanning the node array with the magnetometer probe showed excellent distinction between the simulated ‘sentinel’ and ‘non-sentinel’ nodes (Fig. 6b). The level measured from N1 and N2 remained above the threshold level (SNR of 5.3 and 7.3, respectively), yet the signal from the control node remained below the threshold level.

### *In vivo* measurement of swine lymph nodes

Intraoperative measurements of first-tier draining nodes were performed across two swine. As described above, a 10 mg dose of magnetic tracer was intradermally administered and MRI was used to localise the SLN(s) before surgery. Blue dye was then used to visualise the lymphatics and map the draining from the injection site. After visual identification of the nodes during surgery, the magnetometer probe was used to measure the signal of each node from a range of positions. When the maximum strength signal was identified, the node was measured 3 times at that location and the signal from the node referenced to the surrounding, non-lymphatic tissue. As before, a node was considered positive if the average maximum signal from the node was greater than the probe threshold level (i.e. SNR = 2.0). After excision, the signal from all nodes was measured once more *ex vivo* by comparing the node signal to the local background.

A total of 6 first-tier (also identified as deep popliteal; n = 2 + 4) and 2 second-tier (identified as superficial inguinal; n = 1 + 1) draining nodes were identified with preoperative MRI due to the uptake of the magnetic tracer. During surgery, all first-tier nodes were visually identifiable from the dark brown staining of the magnetic tracer, yet only 4 of these were also identifiable with the blue dye. All first-tier nodes that showed a presence of magnetic tracer on the MRI scans were measured above the probe threshold (Fig. 6a), and confirmed with *ex vivo* measurement. First-tier nodes (N3 and N4) were removed from the hind legs of the first animal, and first-tier nodes (N5a, N5b, N6a, and N6b) were removed from the hind legs of the second animal. The SNRs for N3 – N6b were 6.7, 2.8, 5.2, 8.2, 8.3, and 2.8, respectively. Both of the second-tier nodes identified with MRI were measured with the probe and were above the threshold level (SNR of 3.5 and 4.5, respectively). During surgery, drainage of the blue dye to an additional second-tier node was observed in each animal. These additional nodes did not register a signal when measured with the magnetometer probe, which is supported by the absence of strong negative contrast of these nodes in postinjection MRI scans.

Through Prussian blue staining of node sections, it was found that in all positively measured nodes from the *in vivo* experiment, significant blue staining in afferent trabecula and subcapsular sinuses was observed (Fig. 6c). Likewise, Prussian blue staining of negatively measured nodes (2 second-tier, 5 control nodes) revealed little to no presence of magnetic tracer.

## Discussion

A handheld magnetometer probe based on MTJ technology has been developed. Based on the specific requirement of intraoperative detection of SLNs, the main design criteria were high sensitivity and high spatial resolution. An advanced prototype has been fabricated and validated in a preclinical study using swine as a large animal model. The observed detection limit of 50 μg of magnetic tracer equates to 0.25% of an injected dose (ID) for a clinically relevant 20 mg dose of magnetic tracer. Accumulation of lymphotropic magnetic tracers in SLNs is highly dependent on a number of factors, including the type of cancer, administration route, and the physicochemical properties of the tracers (mostly the hydrodynamic diameter)^19^ For a relatively standard 18 MBq dose of filtered 99 m-technetium sulfur colloid administered intradermally in breast cancer patients, average SLN accumulation is around 2.5 ± 4.9 (s.d.)%^20^.

Based on the magnetometer probe’s longitudinal sensitivity characteristics, the detection limit can be significantly increased if the probe-to-node distance is reduced. Unlike the standard inverse-cubed decay of fields from magnetic dipoles, the decay of the magnetic signal is largely dependent on three factors: the finite volume of the magnetic source, finite size of the sensing volume of the magnetometer probe, and the architecture of the magnetic flux density from the excitation coil. As the sensing volume and magnetic flux density remain constant for the magnetometer probe, then changes to the range will be largely affected by the size/shape of the signal sources (i.e. accumulation in the sentinel node). From the measured data, the magnetic signal decay for a small (6 mm diameter), homogenous concentration of tracer can be modelled with a cubic polynomial function (true for 0 mm < x < 7.5 mm). As a result, and with reference to Fig. 3, the signal measured for this sample at a distance of 4.0 mm is approximately 10% of the signal at 0.0 mm. This indicates the magnetometer probe would be most effective when measuring nodes with tracer uptake concentrated close to the node surface; where it can be measured at very close range and the limit of detection would approach 5 μg of magnetic tracer, or 0.03% of a 20 mg injected dose.

During preclinical large animal studies using swine, uptake of the magnetic tracer in lymph nodes resulted in significant negative contrast on the postinjection MRI scans, providing an anatomically detailed roadmap to assist surgical removal of draining nodes. Across magnetometer probe measurements of first-tier nodes, there was significant variation in the magnetic signal strength, which is likely the result of a combination of factors:

Primarily, variation in lymph node uptake is expected to occur between animals and even between limbs in the same animal. This is demonstrated by the significant variation in size of the 8 first-tier nodes (ranging from 4 mm to 15 mm in largest dimension). Variation in size can have a number of effects, such as variation in the uptake between nodes, and variation in the concentration of the tracer within the node. Another factor affecting the magnetometer probe’s signal magnitude was the varying level of adventitia and adipose tissue surrounding *in vivo* first-tier nodes. This can limit the distance of the probe to the node, and in some cases, the adipose tissue surrounding the node had to be partially or fully resected during surgery to sufficiently expose the node and register a signal with the magnetometer probe.

The presence of tissue in the vicinity of the probe tip introduces a secondary effect: reduction in the magnetic signal *in vivo* due to the negative susceptibility (and hence diamagnetic nature) of surrounding tissue. Using a simple mathematical approximation, more than 0.4 μg of iron oxide nanoparticles would be required per 1.0 cm^3^ of tissue to counteract this diamagnetic effect and be measured by a coil or induction-based magnetometer^21^. However, the impact of diamagnetic tissue on overall signal strength is expected to be low for the MTJ magnetometer probe, given the large dependence of signal strength on distance from the probe tip. Using the measured range (Fig. 3) and spatial resolution (Fig. 4) of the MTJ magnetometer probe, the sensing volume can be approximated with that of a cone (i.e. ~ 0.03 cm^3^). Based on the above calculation, it is approximated that the limit of iron oxide detection due to diamagnetic interference would be 0.01 μg of tracer – a value much lower than the previously calculated 5 μg of tracer required for detection using the MTJ magnetometer probe.

Although the factors above must be considered for the application of the magnetometer probe in the intraoperative setting, variation in signal between nodes *in vivo* was not found to be of consequence. This is because only binary, qualitative (positive / negative) detection is required to locate the SLN; and this can be adequately achieved as long as the signal is above the predetermined threshold value.

By analysing the Prussian blue stained node sections under an optical microscope, the presence of the magnetic tracers in positively measured nodes could be confirmed. Furthermore, while the two negatively measured second-tier nodes could be seen to contain a small level of magnetic tracer, there was a clear distinction in the quantity of magnetic tracer when compared to positively measured first-tier and second-tier nodes.

Besides sensitivity, the spatial resolution of the magnetometer probe is of high importance, especially for malignancies with complex lymphatic drainage such as gastrointestinal and oesophageal cancer. Compared to the spatial resolution of gamma probes and competing magnetometer probes such as the SentiMag (spatial resolution of ~20 mm^22^), the MTJ magnetometer probe demonstrates excellent spatial resolution at just 4.0 mm. From the spatial resolution, the magnetometer probe’s ability to differentiate a SLN signal from an injection site’s background signal can also be determined. For gamma probes, a SLN must be a minimum distance equal to 3 standard deviations of the response curve from an injection site to minimise erroneous readings due to gamma shine-through^23^ For a probe with a spatial resolution of 20 mm, this distance equates to >25 mm, but for the MTJ magnetometer probe, only a distance of >5 mm would be required. Resolution of this order may offer a significant advantage in complex, close-packed lymphatic environments, as further demonstrated in the *ex vivo* spatial resolution measurements presented in Fig. 6b.

In a simulated close-packed array of lymph nodes, the probe demonstrated the ability to accurately distinguish positive (sentinel) nodes from control nodes with near-pinpoint precision. These experiments demonstrate the potential for the MTJ magnetometer probe to be applied beyond breast cancer and melanoma to more complex cancer types such as oesophageal, and other deep tissue cancers of the abdomen.

## Methods

### Magnetometer probe

Highly sensitive (2 nT/Hz^05^ at 100 Hz) single-axis MgO-based MTJ sensors were purchased from MicroMagnetics (STJ-201, Massachusetts, USA). Ultrasonic wedge bonding of aluminium wire between the contact pads of the MTJ sensors and contact pads on the sensor PCB was required due to the small size of each device. Conventional soldering was used for all other electronic components. Electromagnet for the probe tip was machined out of mild steel into a cylindrical bobbin shape 25 mm long, and with a hollow, central aperture 4.0 mm in diameter for the sensor PCB to sit within. Output signals from the probe were acquired via DAQ6009 hardware (National Instruments, Texas, USA) into a custom LabVIEW executable. Detected signals from the magnetic tracer were graphed in real time to provide user feedback during use of the probe. All data were saved to a data file for computational analysis.

### Measuring magnetic particles

Iron oxide nanoparticles with a dextran coating were purchased from Chemicell (*FluidMag-DX*, Berlin, Germany) for use in magnetic phantoms during *in vitro* characterisation, and as a lymphotropic tracer in large animal experiments. The hydrodynamic diameter of the tracer was measured using dynamic light scattering to be 28 nm with a distribution width of 20 nm. Changes in tracer size distribution were measured over a wide range of pH levels (1.5 to 11), as well as in saline and phosphate buffer solution, with no significant changes to stability evident. Two types of dried magnetic phantom were constructed for use with the magnetometer probe: nonpoint-source (NPS) and ‘point-source’ (PS) phantoms. NPS phantoms were used for sensitivity measurements, and were constructed by cementing glass coverslips to the face of 4.0 mm thick polyoxymethylene discs with a 5.0 mm central aperture, creating a small well. Controlled volumes of magnetic tracer were dried in the wells, creating a thin, uniform layer of magnetic particles at the bottom of the well. PS phantoms were used for *in vitro* spatial resolution measurements and were constructed by creating a 3.0 mm diameter aperture in a 1.5 mm thick polycarbonate disk, and laminating it to another equally sized disk without an aperture, creating a smaller volume well. Similarly, quantities of magnetic tracer were dried in the well, but due to the small aperture size and viscosity of the tracer, the result was a volume of dried particles of similar dimensions to the well, rather than a thin layer at the bottom. In order to determine the quantity of tracer in each phantom, 0.1 mL of the stock was dissolved in 36% HCl solution, which was added to an aqueous solution of K_4_[Fe(CN)_6_]. After 3 minutes, the UV-Vis absorption spectrum of the solution was measured from 500 – 800 nm, and the intensity of the spectrum was compared to a calibration curve from known concentrations of FeCl_3_ solution to determine the iron content. For all measurements, the uncertainty in a signal was taken as the ± 1 standard deviation of variation in the measured background signal, and the noise level as ± 3 standard deviations. The noise level calculated in this manner was used to find the SNR.

### Large animal experiments

Pre-clinical large animal experiments using swine were carried out in accordance with the National Health and Medical Research Council’s Australian code of practice for the care and use of animals for scientific purposes. All experimental protocols using animals were approved by both Adelaide University and National Imaging Facility animal ethics. In total, three female Large White swine (31 – 35 kg) were used across two preclinical studies. For all the swine studies, the magnetic tracer was prepared to a 20 mg/mL concentration in 0.9% saline, and injected without further purification. Patent Blue V dye was prepared according to manufacturer’s instructions and used to visually identify the lymphatic vessels leading to draining lymph nodes. T1 sequences were acquired using the following parameters: TR/TE 1530 ms/4.08 ms, flip angle 15°, FOV phase 75%, 5 mm slice thickness (axial and coronal). T2* sequences were acquired using: TR/TE of 155 ms/10 ms, flip angle of 90°, FOV phase of 75%, 5 mm slice thickness (axial and coronal). Excised nodes were stored in a 10% formalin solution with any surrounding adventitia or adipose tissue left intact, unless sectioned for Prussian blue staining. If used for staining, nodes < 5 mm in size were halved, and nodes > 5 mm were sliced into 3 – 5 mm sections and embedded in paraffin wax blocks. Two slices of 4 μm thickness were taken from each embedded node section for haematoxylin and eosin (H&E) and Prussian blue staining, respectively.

## Additional Information

**How to cite this article**: Cousins, A. *et al*. Novel Handheld Magnetometer Probe Based on Magnetic Tunnelling Junction Sensors for Intraoperative Sentinel Lymph Node Identification. *Sci. Rep*. **5**, 10842; doi: 10.1038/srep10842 (2015).
